# Acute Encephalopathy in Children From Muzaffarpur, Bihar, India, and the Potential Role of Ambient Heat Stress-Induced Mitochondrial Dysfunction

**DOI:** 10.7759/cureus.37073

**Published:** 2023-04-03

**Authors:** Arun K Singh, Manoj Jhalani, Sunil K Shahi, Rita Christopher, Bhartendu Kumar, Manoja K Das

**Affiliations:** 1 Neonatology, All India Institute of Medical Sciences, Jodhpur, Jodhpur, IND; 2 National Health Mission, Ministry of Health and Family Welfare, Government of India, New Delhi, IND; 3 Medical Administration, Sri Krishna Medical College and Hospital, Muzaffarpur, IND; 4 Neurochemistry, National Institute of Mental Health & Neuro Sciences, Bengaluru, IND; 5 General Surgery, Sri Krishna Medical College and Hospital, Muzaffarpur, IND; 6 Public Health, The INCLEN Trust International, New Delhi, IND

**Keywords:** bihar, muzaffarpur, heat stress, mitochondrial dysfunction, children, acute encephalopathy

## Abstract

Background: Periodic outbreaks of acute encephalopathy in children have been reported from Muzaffarpur, Bihar, India. No infectious cause has been identified for this. This study presents the clinical and metabolic profile of children hospitalized with acute encephalopathy and the potential role of ambient heat stress.

Methods: This cross-sectional study included children (<15 years) with acute encephalopathy admitted from April 4, 2019, to July 4, 2019. The clinical and laboratory investigations included infections, metabolic abnormalities, and muscle tissue analysis. The children who had metabolic derangements but no infectious cause were labeled as acute metabolic encephalopathy. The descriptive analysis summarized the clinical, laboratory, and histopathology findings, and their association with the ambient heat parameters was explored.

Results: Out of the 450 children hospitalized (median age, four years), 94 (20.9%) died. Children had early morning onset (89%), seizures (99%), fever (82%), hypoglycemia at admission (64%), raised aminotransferases (60%), and high blood urea (66%). Blood lactate (50%), lactate dehydrogenase (84%), pyruvate (100%), ammonia (32%), and creatinine phosphokinase (69%) were raised. Viral marker tests were negative. The patients had abnormal metabolic markers like decreased blood-free carnitine, elevated blood acylcarnitines, and elevated urinary lactate, oxalate, maleate, adipate, and fatty acid metabolites. Blood carnitine and acylcarnitine levels normalized in 75% of the patients treated with carnitine and coenzyme-Q. Muscle tissues showed megamitochondria on electron microscopy and reduced respiratory enzyme complex-I activity. A significant correlation between the number of admissions and ambient heat indices was observed.

Conclusions: The findings suggest secondary mitochondrial dysfunction as a possible mechanism for acute encephalopathy in children from Muzaffarpur, Bihar, and ambient heat stress as a possible risk factor.

## Introduction

Seasonal outbreaks of unexplained acute encephalopathy in children have been reported in Muzaffarpur district (Bihar, India) in 2005, 2009, 2011, 2012, 2013, 2014, and 2019 with high case fatality (27-63%) [1-5]. There have been several efforts in the past to document the aetiology of periodic acute encephalopathy outbreaks in children but no infectious aetiology for these has been identified in the past [1-5]. The epidemiology of these acute encephalopathy outbreaks, the profile of the children (majority of the children aged three to seven years), clinical features (early morning onset, seizures, altered sensorium, fever, and hypoglycemia), and period of the outbreaks (May-June months) have remained unchanged over these years [1-5]. 

In absence of definite infectious aetiology, several alternate hypotheses have been proposed including pesticides, toxins, lychee fruit, and heat exposure for the outbreaks [2-6]. The 2014 outbreak investigation reported lychee toxins, methylene-cyclo-propyl-glycine (MCPG), and methylene-cyclo-propyl-acetic-acid (MCPA), as the potential causes [5-7]. The concentration of these encephalopathy cases in the Muzaffarpur district alone while lychee is cultivated in other districts doesn’t satisfy the explanation. 

Heat stress exposure in animals and humans has been reported to cause metabolic derangements and mitochondrial dysfunctions, which can explain the majority of the biochemical derangements reported during the 2014 outbreak [5-7]. Also, the changes in the central nervous system and neuronal level could be explained by heat stress and mediated mitochondrial derangements [8-14]. 

During the acute encephalopathy outbreak in April 2019, the Ministry of Health and Family Welfare (MoHFW), Government of India, deputed a team of specialists to investigate the outbreak. This article describes the acute encephalopathy outbreak investigation findings including the clinical and laboratory profiles of the patients and explored the association with possible risk factors like ambient heat stress.

## Materials and methods

This was a cross-sectional study conducted at a government tertiary care hospital, Sri Krishna Medical College Hospital, in Muzaffarpur, Bihar, India.

Participants and evaluation

All the children aged <15 years who were admitted to the hospital from April 4 to July 4, 2019, with acute encephalopathy were included. Acute encephalopathy was characterized by acute onset of change in mental status (confusion, disorientation, inability to talk, or coma) and/or seizure with/without fever. Patients admitted with simple febrile seizure, any underlying neurological, metabolic disorders and organ (renal or hepatic) dysfunction, and other associated systemic infections were excluded.

The patients were managed according to the protocol for acute encephalitis case management and the required clinical and laboratory tests were carried out. For all patients, blood glucose and haemogram were done. Whenever feasible, renal function tests, serum electrolytes, liver function tests, cerebrospinal fluid (CSF) analysis for cytology, bacterial culture, and chemistry (total protein and glucose) were done. The serum and CSF specimens were tested for IgM antibodies against Japanese encephalitis (JE), dengue, measles, mumps, rubella, Chandipura, and enteroviruses using enzyme-linked immunosorbent assay, whenever feasible. For some patients, urine, dried blood spot (DBS), and plasma samples were collected within six hours of admission and tested for metabolic abnormalities and ketone bodies. The urine samples were tested for metabolic abnormalities by gas chromatography-mass spectroscopy (GC/MS) methods. The plasma and DBS samples were tested using the tandem mass spectroscopy (MS/MS) method for free carnitine and acylcarnitines. For 10 patients, the plasma and DBS samples were tested before and after five to six days of therapy with carnitine (50mg/kg/day) and coenzyme-Q (10mg/kg/day).

Whenever feasible, a brain CT scan and electroencephalogram were done. Muscle biopsies done in two patients were subjected to histopathology, immunohistochemistry, electron microscopy, and respiratory chain enzyme (spectrophotometry) analyses. The patients of acute encephalopathy with no infectious aetiology identified and some metabolic abnormality like blood sugar (<50mg/dL), blood gas (pH <7.38, bicarbonate (HCO3) <22mmol/L, anion gap >12), plasma ammonia (>100mcg/dL), blood lactate (>22mg/dL), plasma ketone (>1mmol/L), plasma amino acids, liver function tests (aspartate aminotransferase (AST) >40IU/L, alanine transaminase (ALT) >40IU/L, ALP >240IU/L), and urinary organic acids were considered as acute metabolic encephalopathy.

Data collection, management, and analysis

For the cases, anonymized data on demography, clinical, investigations, treatment and outcome were prospectively collected and abstracted. The biochemical tests were interpreted in comparison to the age-appropriate reference values. The ambient heat and meteorological data including daily temperatures (maximum, minimum, average), humidity, wind speed, rainfall, and heat index for the period of April to July 2019 were collected retrospectively from www.worldweatheronline.com with due permission. The data were analyzed with Stata version 15.0 (Released 2017; StataCorp LLC, College Station, Texas, USA). Descriptive analysis findings were presented as numbers, proportions, means and standard deviations and median and interquartile range (IQR), as appropriate. Pearson’s correlation analysis explored the correlation between daily acute encephalopathy caseload (dependent variable), and mean meteorological parameters for the periods, previous one, two, three, and seven days (independent variable).

Ethical aspects

The Institute Ethics Committee, Sri Krishna Medical College, Muzaffarpur, Bihar, India, reviewed the protocol and exempted it from obtaining consent from patients (approval number: 17/20 (IEC, dated 31/01/2020). The de-identified data for the patients admitted with acute encephalitis syndrome (AES) were collected from the hospital records.

## Results

**Figure 1:**
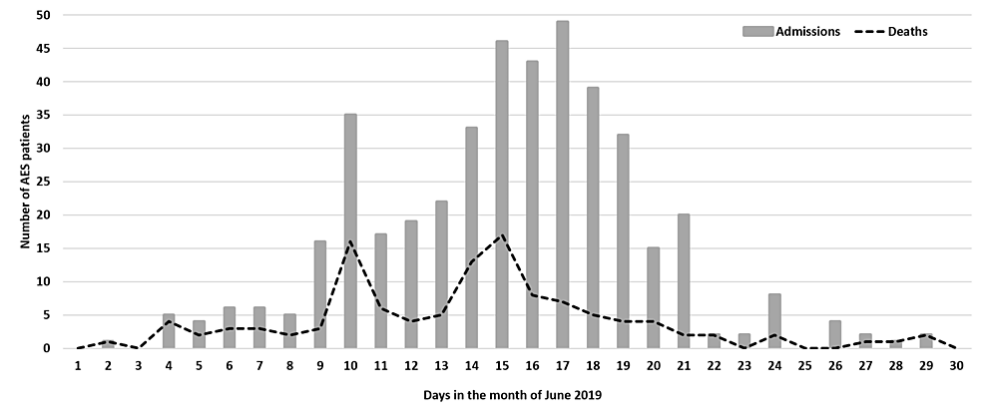
The pattern of admissions and deaths among the children with acute encephalopathy in Muzaffarpur, Bihar, India, in June 2019 Note: The day indicates the date of June month in the year 2019. Admissions indicate the number of patients with acute encephalopathy admitted to the hospital on the day. Deaths indicate the number of acute encephalopathy patients who died on the day. AES: acute encephalopathy syndrome

According to National Center for Disease Control (NCDC), India, the outbreak lasted from April 4 to July 4, 2019, and 842 encephalopathy cases were reported from Bihar with 168 (20%) deaths. The majority of the cases were from Muzaffarpur district (544 cases, 99 deaths) followed by East Champaran (106 cases, 24 deaths), Sitamarhi (30 cases, 11 deaths), and Vaishali (73 cases, 15 deaths) districts; the remaining (89 cases, 19 deaths) were from other seven districts. For the study, we could collect information for 454 children. Four patients with the diagnosis of JE were excluded. The data for 450 cases were analyzed, out of which 94 (21%) died. In Muzaffarpur district, 70% of these cases were from three blocks: Meenapur, Mushahri, and Kanti. Almost all (n=447, 99%) cases occurred during June 2019, from June 9-21, 2019, with the peak during June 15-17, 2019 (Figure 1).

The expert team was present for two weeks at the hospital and facilitated better clinical record-keeping. Detailed clinical and investigation data for 150 patients could be retrieved from the case records.

Clinical presentations

**Figure 2:**
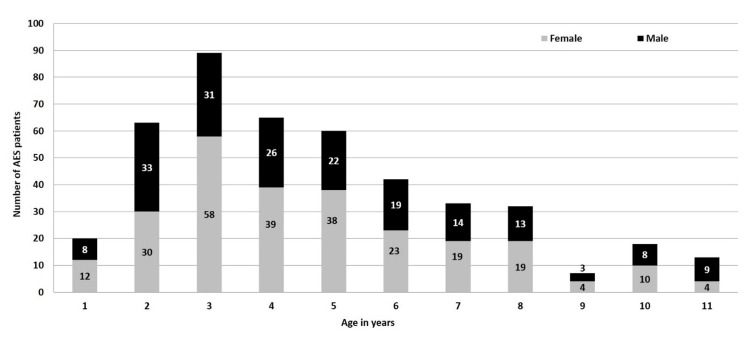
The age and gender distribution of the children admitted with acute encephalopathy in Muzaffarpur, Bihar, India, during 2019 Note: Age indicates the ages of the AES children admitted to the hospital. Number indicates the number of AES cases admitted to the hospital. AES: acute encephalopathy syndrome

The median age of these children was 48 months (IQR: 36-72 months) and 261 (58%) were girls. The majority (63%) of these children were aged two to five years (Figure 2).

Table 1 shows the sociodemographic and clinical features and outcomes of these children. Almost all (98%) had generalized tonic-clonic seizures with early morning onset (89%) and fever (82%; mean temperature 100.2^0^C and SD 1.5^0^C). Assisted ventilation was provided to 13/91 (14%) patients and several could not be supported due to the non-availability of ventilators.

**Table 1:** Characteristics, clinical features, and outcomes of children with acute encephalopathy in Muzaffarpur, Bihar, India, in 2019 ^†^ n indicates the observed value and N indicates the total valid responses/data available; N indicates the denominator for % estimation. ICP: intracranial pressure; IQR: interquartile range; LAMA: left against medical advice

Parameters	Observations n/N^†^ (%)
Gender	
Boys	191/450 (42.5)
Girls	259/450 (57.5)
Age groups	
Median (IQR) in months	48 (36-72)
< 1 year	19/449 (4.2)
>1-5 years	282/449 (62.8)
>5-10 years	135/449 (30.0)
>10 years	13/449 (2.9)
Clinical features	
Morning symptoms	100/113 (88.5)
Seizure	121/123 (98.4)
Fever	101/124 (81.5)
Pain abdomen	4/114 (3.5)
Vomiting	12/117 (10.3)
Shock	44/118 (37.3)
Raised ICP	36/93 (38.7)
Timing of presentation to hospital	
00:00- 08:00 Hrs	261/450 (58.0)
08:00-12:00 Hrs	81/450 (18.0)
12:00-18:00 Hrs	58/450 (13.0)
18:00-00:00 Hrs	50/450 (11.0)
Duration of hospital stay	
Median (IQR) in days	3 (1, 4)
≤1 day	129/450 (28.7)
2-5 days	271/450 (60.2)
>5 days	50/450 (11.1)
Patient outcomes	
Discharged	336/450 (74.7)
Died	94/450 (20.9)
Referred/LAMA	20/450 (4.4)

Investigation findings

Table 2 summarises the investigation findings in the patients. At admission, blood sugar was <50 mg/dL for 51.8% (59/114) of the patients, and 11% (11/114) and 18% (20/114) had blood sugar levels >150 mg/dL and 101-150 mg/dL, respectively, as many of the patients received dextrose injection and/or intravenous fluids at first healthcare contact before referral. Abnormal ALT, AST, and blood urea were observed in 60%, 57%, and 66% of the patients, respectively. Abnormal serum lactate, lactate dehydrogenase (LDH), pyruvate, ammonia, creatinine phosphokinase (CPK), and CPK-myocardial band (CPK-MB) isoenzyme levels were observed in 50-100% of the patients tested (Table 2). Metabolic acidosis (13/28, 53%), low HCO3 (23/28, 82%), and high anion gap (100%) were observed in 28 patients (venous blood gas). CSF samples tested in 10 patients were negative for rubella, measles, mumps, and Chandipura viruses. Scrub typhus serology was negative in 40 patients tested.

**Table 2:** Laboratory investigation findings for the children with acute encephalopathy in Muzaffarpur, Bihar, India, in 2019 ^† ^n indicates the observed value and N indicates the total valid responses/data available; N indicates the denominator for % estimation. ^‡^ Urine ketone was tested using the dipstick method AST: aspartate aminotransferase; ALT: alanine aminotransferase; ALP: alkaline phosphatase; CPK: creatinine phosphokinase; CPK-MB: creatinine phosphokinase-myocardial band; CSF: cerebrospinal fluid; INR: international normalized ratio; LDH: lactate dehydrogenase; PT: prothrombin time; TLC: total leukocyte count

Parameters	Findings n/N^†^ (%)	Median (IQR)	Range (min, max)
Hematological parameters			
Anemia (Haemoglobin <110 g/L)	107/150 (71.3)	10.3 (9.7, 11.1)	(5.3, 13.8)
Abnormal TLC (<4000 or >11000/cmm)	108/150 (72.1)	13,800 (10,575, 19,185)	(2,900, 29,200)
Blood metabolic parameters			
Blood glucose (at admission) <50mg/dL (<2.8mmol/L)	59/114 (51.8)	47.5 (35, 105)	(20,390)
Serum bilirubin >2 IU/L	1/149 (0.7)	0.8 (0.7, 0.9)	(0.4,2.1)
Serum AST >40 IU/L	86/150 (57.3)	47 (33, 69)	(18,282)
Serum ALT >40 IU/L	90/150 (60.0)	46 (32, 69)	(18,204)
Serum ALP >240 IU/L	27/149 (18.1)	172 (149, 214)	(49,473)
Both AST and ALT >40 IU/L	73/150 (48.7)	-	-
PT >14 seconds	4/4 (100)	21.45 (18.95, 26)	(17,30)
INR >2	6/12 (50.0)	2 (1.75, 2.5)	(1.3,3.5)
Serum albumin <3.5g/dL (<35g/L)	23/145 (15.9)	3.9 (3.7,4.2)	(2.8,5)
Blood urea >40 mg/dL (>6.6 mmol/L)	98/148 (66.2)	49 (36.5, 60)	(11, 102)
Serum creatinine >1 mg/dL (>88.4µmol/L)	2/148 (1.4)	0.7 (0.6, 0.7)	(0.4, 1.5)
Serum ammonia >100mcg/dL (>58.72 µmol/L)	14/44 (31.8)	83.45 (67.9, 118.1)	(44.3, 266)
Serum lactate >22mg/dL (>2.44 mmol/L)	9/18 (50.0)	21.9 (16.3, 29.9)	(8, 334.9)
Serum LDH>600 U/L	26/31 (83.9)	1408 (718.3, 2604)	(330, 4074)
Serum pyruvate>1.4mg/dL (>0.16 mmol/L)	18/18 (100)	80.25 (62, 112.9)	(33, 187.3)
Serum CPK >300U/L	18/26 (69.2)	552.5 (253.3, 1054)	(44.6, 10805)
Serum CPK-MB >3µg/L (0.05 ukat/L)	16/16 (100)	127.9 (63.5, 186.5)	(13.9, 330)
Serum ketone >1mmol/L	5/7 (71.4)	2.6(0.3,4.5)	(0.3, 7.8)
Urine metabolic parameter			
Urine ketone positive ^‡^	8/9 (88.8)	-	-
CSF findings			
CSF cells >5/cc	5/10 (50.0)	6 (4, 10)	(4, 50)
CSF sugar<40 mg/dL (<2.22 mmol/L)	1/10 (10.0)	53.5 (52, 59)	(31, 67)
CSF protein >80mg/dL	5/10 (50.0)	70 (46, 90)	(44, 96)

Out of the eight urine samples analyzed by GC/MS method, the majority had elevated lactate, oxalate, maleate, adipate, and fatty acid metabolites (Table 3).

**Table 3:** The urinary metabolite profiles in the children with acute encephalopathy in Muzaffarpur, Bihar (by GC/MS method) ^a^ n indicates the number of abnormal values observed and N indicates the total test results available NA: Not available (the IQR for parameters with <3 values could not be derived); GC/MS: gas chromatography/mass spectrometry; 2HIV: 2-hydroxyisovalerate; 2HIC: 2-hydroxyisocaproic acid; 3HIV: 3-hydroxyisovalerate; 3HG: 3- hydroxyglutarate; 3HP: 3-hydroxypropionic acid; 2M3HB: 2methyl-3- hydroxybutrate; 3HB: 3-hydroxybutrate; 3H-C12-DC: 3-hydroxy-dodecanedioylcarnitine; 4HB: 4-hydroxybutrate; PA: Propionic acid; 4HPA: 4-hydroxypropionic acid; 4HPL: 4-hydroxyphenyllactate; MMA: Methylmalonic acid; 2,3-DH-2-MB: 2,3-dihydroxy-2-methylbutyrate; IQR: interquartile range

Metabolites	Abnormal values, n/N^a^	Observed value Median (IQR)	Number of times rise Median (IQR)
Lactate	7/7	183.7 (114-712.9)	338.1 (202.5-786)
Oxalate	3/3	7.0 (1.3-116.2)	886.6 (168.2-14535.5)
Fumarate	2/2	115 (NA)	4608.5 (NA)
Glycerol	1/1	171.0 (NA)	1155.734 (NA)
Glycerate	1/1	577.8 (NA)	9794.8 (NA)
Glutarate	2/2	8.6 (NA)	666.8 (NA)
Malate	5/5	3.7 (0.9-49.6)	755.6 (232.4- 49661.3)
Succinate	2/2	5.3 (NA)	246.7 (NA)
Me-succinate	1/1	1.0 (NA)	207.1 (NA)
Methylsuccinate	1/1	0.5 (NA)	102.84 (NA)
3Hydroxysebacate	1/1	0.8 (NA)	208.7 (NA)
Adipate	5/5	6.9 (3.2-49.1)	82.4 (38.6- 447.8)
Suberate	2/2	0.1 (NA)	16.8 (NA)
Fumarate	1/1	2.2 (NA)	101.8 (NA)
Sebacate	1/1	0.3 (NA)	12.1 (NA)
2HIV	4/4	1.5 (1.1-5.3)	222.6 (83.6- 887.5)
2HIC	2/2	0.4 (NA)	466.3 (NA)
3HIV	2/2	61.3 (NA)	183.3 (NA)
3HG	1/1	0.4 (NA)	52.4 (NA)
3HP	1/1	2.6 (NA)	39.2 (NA)
2M3HB	1/1	49.8 (NA)	664.4 (NA)
3HB	5/5	21.0 (2.8- 606.2)	1053.5 (140.6- 29770.7)
3H-C12-DC	1/1	0.1 (NA)	111.9 (NA)
4HB	1/1	1.1 (NA)	1153 (NA)
PA	1/1	0.9 (NA)	983.7 (NA)
4HPA	1/1	10.2 (NA)	40.5 (NA)
4HPL	2/2	3.9 (NA)	49.7 (NA)
MMA	3/3	2.8 (0.1-3.2)	352.6 (86.9- 408.7)
2,3-DH-2-MB	3/3	2.4 (1.5- 9.2)	(NA)

Deranged plasma/DBS carnitine and acylcarnitine levels were found in 85.7% (30/35) patients before carnitine/coenzyme-Q therapy and in 40% (12/30) after therapy (Table 4).

**Table 4:** The blood carnitine and acylcarnitines metabolite profiles in the children with acute encephalopathy in Muzaffarpur, Bihar (by MS/MS method) Note: The plasma acylcarnitines were estimated by tandem mass spectrometry (MS/MS).

Carnitine/Acylcarnitine	Pre-therapy only (n=35)	Post-therapy only (n=30)	Pre- and post-therapy levels for the same child	
			Pre-therapy (n=10)	Post-therapy (n=10)
C0, n (%)	18 (51.4)	4 (13.3)	7 (70)	1 (10)
C2, n (%)	13 (37.1)	3 (10)	4 (40)	0 (0)
C3, n (%)	3 (8.6)	0 (0)	0 (0)	0 (0)
C3DC, n (%)	18 (51.4)	10 (33.3)	7 (70)	1 (10)
C4DC, n (%)	3 (8.6)	3 (10)	0 (0)	0 (0)
C5DC, n (%)	1 (2.9)	0 (0)	0 (0)	0 (0)
C6, n (%)	19 (54.3)	0 (0)	5 (50)	0 (0)
C6DC, n (%)	10 (28.6)	0 (0)	2 (20)	0 (0)
C5, n (%)	12 (34.3)	2 (6.7)	3 (30	0 (0)
C4, n (%)	2 (5.7)	0 (0)	0 (0)	0 (0)
C10, n (%)	10 (28.6)	1 (3.3)	2 (20)	1 (10)
C10:1, n (%)	10 (28.6)	0 (0)	0 (0)	0 (0)
C10:2, n (%)	0 (0)	0 (0)	0 (0)	0 (0)
C12, n (%)	15 (42.9)	0 (0)	5 (50)	0 (0)
C12:1, n (%)	10 (28.6)	0 (0)	5 (50)	0 (0)
C14, n (%)	16 (45.7)	2 (6.7)	5 (50)	0 (0)
C14-OH, n (%)	0 (0)	0 (0)	0 (0)	0 (0)
C14:1, n (%)	15 (42.9)	7 (23.3)	5 (50)	3 (30)
C16, n (%)	14 (40)	0 (0)	3 (30)	0 (0)
C16-OH, n (%)	0 (0)	0 (0)	0 (0)	0 (0)
C16:1, n (%)	19 (54.3)	0 (0)	6 (60)	0 (0)
C18, n (%)	5 (14.3)	0 (0)	2 (20)	0 (0)
C18-OH, n (%)	0 (0)	0 (0)	0 (0)	0 (0)
C18:1, n (%)	5 (14.3)	1 (3.3.)	1 (10)	0 (0)
C18:1-OH, n (%)	0 (0)	0 (0)	0 (0)	0 (0)
C18:2, n (%)	0 (0)	0 (0)	0 (0)	0 (0)
C8, n (%)	12 (34.3)	0 (0)	4 (40)	0 (0)
C8:1, n (%)	1 (2.9)	0 (0)	0 (0)	0 (0)
C4-OH, n (%)	18 (51.4)	0 (0)	7 (70)	0 (0)
C5-OH, n (%)	1 (2.9)	0 (0)	0 (0)	0 (0)
C5:1, n (%)	0 (0)	0 (0)	0 (0)	0 (0)
Acylcarnitines abnormal, n (%)	24 (68.6)	14 (46.7)	4 (40)	7 (70)
Carnitine/ Acylcarnitines abnormal, n (%)	30 (85.7)	18 (60)	9 (90)	4 (40)

**Figure 3:**
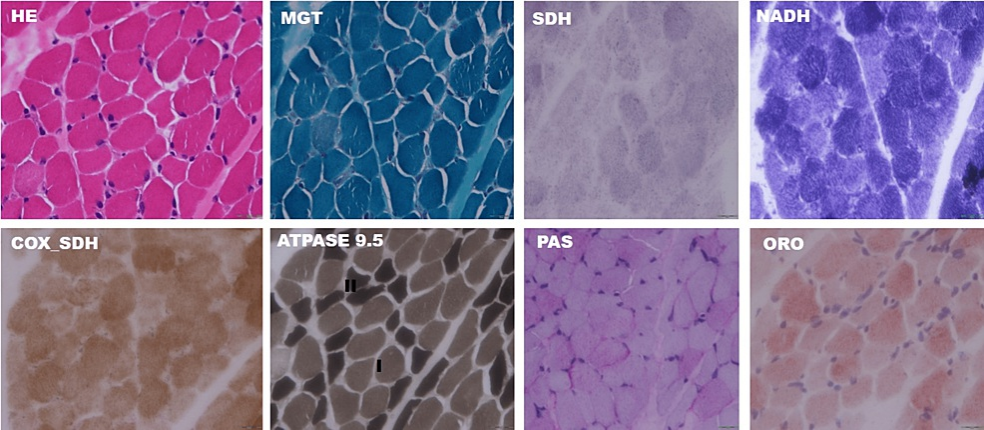
Muscle histopathology and electron microscopy findings from a patient with acute encephalopathy Histochemical stains shows Type II (dark stained) fibre atrophy on ATPase 9.5 stain. There is no evidence of mitochondrial pathology or storage material (glycogen or lipid). H&E: haematoxylin & eosin; MGT: modified Gomori's trichrome; NADH-TR: nicotinamide adenine dinucleotide tetrazolium reductase; ORO: Oil Red O; PAS: periodic acid–Schiff; SDH: succinic dehydrogenase; SDH-COX: succinic dehydrogenase-cytochrome oxidase

**Figure 4:**
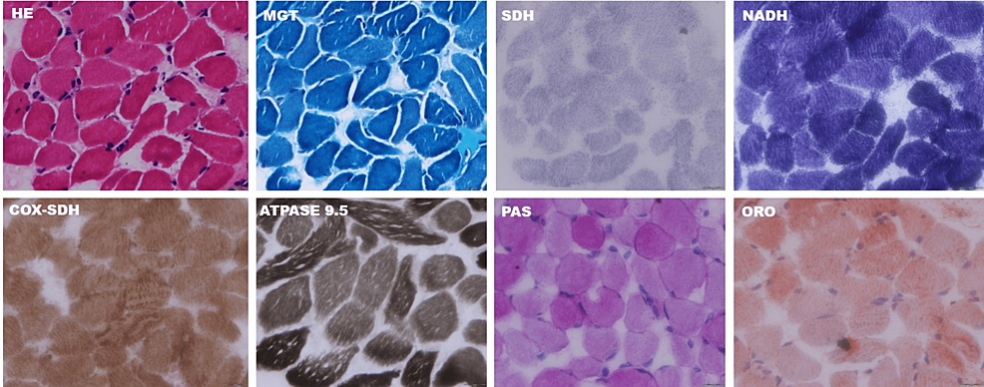
Muscle histopathology and electron microscopy findings from another patient with acute encephalopathy Histochemical stains shows mild fibre variations. There is no evidence of mitochondrial pathology or storage material (glycogen or lipid). H&E: haematoxylin & eosin; MGT: modified Gomori's trichrome; NADH-TR: nicotinamide adenine dinucleotide tetrazolium reductase; ORO: Oil Red O; PAS: periodic acid–Schiff; SDH: succinic dehydrogenase; SDH-COX: succinic dehydrogenase-cytochrome oxidase

**Figure 5:**
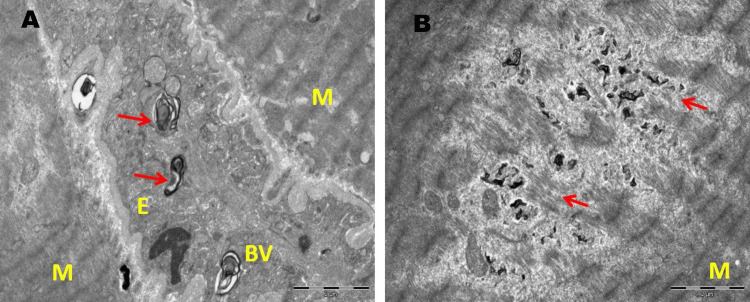
Electron microscopy findings of muscle from a patient with acute encephalopathy The skeletal muscle tissue shows presence of myeloid structures (shown with arrows) within the endothelial cells of the blood vessels (panel A) and within the myofibres (panel B). BV: blood vessel; E: endothelial cell; M: myofibre

**Figure 6:**
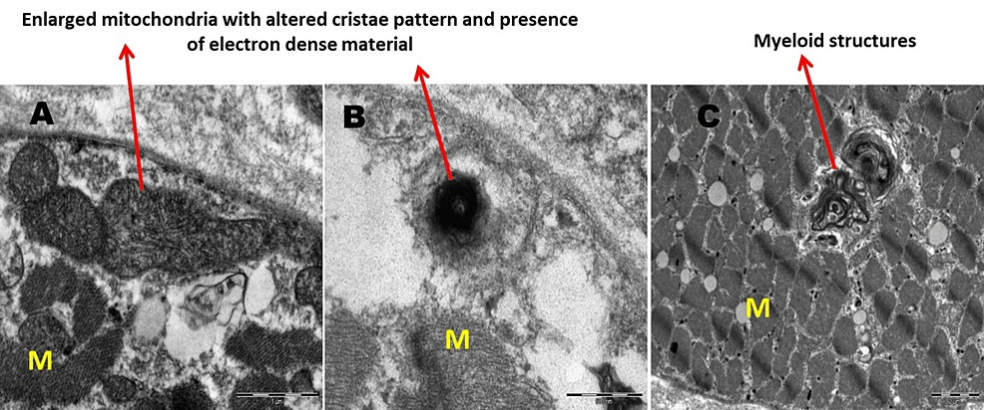
Electron microscopy findings of muscle from another patient with acute encephalopathy The skeletal muscle tissue shows a few enlarged mitochondria with altered cristae pattern (panel A), presence of electron dense material (panel B), presence of myeloid structures and distortion of myofilamentous pattern in a few fibres (panel C). Detailed histopathology findings: There is mild variation in fibre size. Sarcolemma is normal. Nuclei are peripherally placed.  Intranuclear inclusions are not seen. Myofilamentous pattern is maintained in the majority of the fibres while a few show distortion with clumped Z-band material. The morphology of mitochondria is altered in a few fibres. The alteration includes mildly enlarged mitochondria with altered cristae patterns and the presence of electron-dense material. There is no evidence of glycogen or lipid accumulation. Intermyofibrillar and subsarcolemmal presence of myeloid structures are noted. The tubular system appears normal. There are no inflammatory cells. Blood vessels show thickened basement membranes, normal mitochondria, and a few myeloid structures. M: myofibre

Muscle histopathology (two cases) revealed fibre size variation and electron microscopy showed enlarged mitochondria, altered cristae pattern, presence of electron-dense material and clumped z-band (Figures 3-6).

Grossly, low respiratory enzyme complex-I activity (0.07-0.08 µmols, 24-28% of control mean) was observed for the two patients (Table 5).

**Table 5:** Muscle respiratory chain enzyme complex test results in the patients with acute encephalopathy Units of measurement: ^a^ µmols dichlorophenolindophenol (DCIP) reduced.min-1.mg protein-1.unit citrate synthase-1; ^b^ µmols Cytochrome C reduced.min-1.mg protein-1.unit citrate synthase-1

Parameters	Patient 1		Patient 2		Reference mean (range)
	Result	% of control mean (%)	Result	% of control mean (%)	
Complex I	0.07	24.01	0.08	27.89	0.30 (0.107-0.499)^a^
Complex II	0.25	58.12	0.32	77.03	0.42 (0.227-0.649)^a^
Complex III	0.98	218.8	0.73	163.39	0.45 (0.161-0.609)^b^
Complex IV	0.59	57.11	0.71	69.51	1.02 (0.264-1.78)^b^

Case management

**Figure 7:**
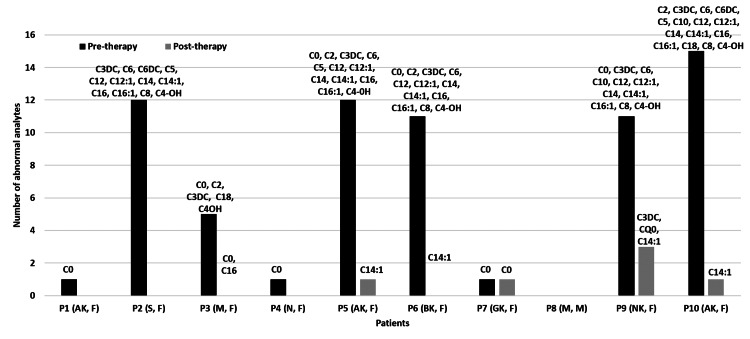
Changes in blood carnitine and acylcarnitine levels among the children with acute encephalopathy before and after carnitine and coenzyme-Q therapy Pre-therapy indicates the levels of metabolites before the carnitine and coenzyme-Q therapy in the patients. Post-therapy indicates the levels of metabolites after/on treatment with the carnitine and coenzyme-Q in the patient. The compounds denoted as C with numbers indicate the acylcarnitines before and after carnitine and coenzyme-Q therapy in the patients. C0-C16 indicate the acylcarnitine metabolites; P1-P10 indicates the patients. M: male, F: female

Recurrent episodes of hypoglycemia were observed in 23% (34/150) of the patients when the dextrose infusion replacement was delayed or the infusion rate was tapered (four of them expired). Carnitine (50 mg per kg/day) and coenzyme-Q (10 mg per kg/day) were given to 37 patients. Repeat samples from 10 patients showed normalization of free carnitine (n=9) and acylcarnitines (n=7) levels after five to six days of therapy (Figure 7). In these patients, the serum CPK, CPK-MB, LDH, ammonia, lactate, pyruvate levels normalized and urinary ketone disappeared. At discharge, 72% (108/150) of the patients had neurological deficits.

Sociodemographic parameters

A history of encephalopathy in siblings was noted in 5% (5/103) of patients. Parents gave a history of lychee intake within 24 hours before admission in 27% (28/103) of the cases. Most (91/106; 86%) of these patients belonged to poorer families and 45% (68/150) were underweight for age, although the detailed anthropometry could not be documented.

Relationship with ambient heat stress and meteorological parameters

The caseload of acute encephalopathy in children increased when the maximum ambient temperature crossed 40^0^C and sustained for two to three days, and declined with the drop in the temperature and onset of rain. Significant correlations between the acute encephalopathy caseload/admissions and weather parameters for the previous one, two, three, and seven days during the year 2019 were observed; positive with maximum (r=0.318 to 0.343) and minimum (r=0.371 to 0.395) ambient temperatures, and heat index (r=0.428 to 0.446) (p values <0.001). No significant correlation with rainfall/precipitation, humidity, wind speed, and sunshine hours was noted (Table 6).

**Table 6:** Pearson correlation analysis between daily acute encephalopathy caseload and mean meteorological parameters for previous one, two, three, and seven days CI: confidence interval; hrs: hours; Kmph: kilometer per hour; mm: millimeter * Correlation is significant at the 0.05 level (2-tailed); ** Correlation is significant at the 0.01 level (2-tailed).

Reference period for meteorological parameters	The Pearson correlation coefficient (r) for parameters (95% CI)						
	Maximum temperature (^0^C)	Minimum temperature (^0^C)	Humidity (%)	Wind speed (Kmph)	Precipitation (mm)	Sunshine duration (hrs)	Heat index
Previous one day (24 hours)	0.318^**^ (0.148, 0.469)	0.371^**^ (0.207, 0.515)	-0.115 (-0.287, 0.064)	0.120 (-0.059, 0.291)	-0.119 (-0.291, 0.060)	0.163 (-0.015, 0.331)	0.428^** ^ (0.270, 0.563)
Previous two days (48 hours)	0.325^**^ (0.168, 0.471)	0.384^**^ (0.227, 0.520)	-0.116 (-0.289, 0.069)	0.131 (-0.063, 0.298)	-0.121 (-0.295, 0.063)	0.164 (-0.017, 0.334)	0.432^** ^ (0.272, 0.565)
Previous three days (72 hours)	0.332^**^ (0.172, 0.475)	0.388^**^ (0.232, 0.526)	-0.118 (-0.291, 0.072)	0.138 (-0.065, 0.302)	-0.124 (-0.299, 0.066)	0.166 (-0.019, 0.337)	0.441^** ^ (0.279, 0.570)
Previous seven days (168 hours)	0.343^**^ (0.188, 0.482)	0.395^**^ (0.241, 0.535)	-0.120 (-0.293, 0.076)	0.142 (-0.069, 0.309)	-0.128 (-0.302, 0.069)	0.169 (-0.022, 0.342)	0.446^** ^ (0.282, 0.576)

Regression analysis also documented a significant association between the acute encephalopathy caseload/admissions and the meteorological parameters for the previous one to seven days during 2019; positive association with the maximum (coefficient=0.774 to 0.896) and minimum (coefficient=1.251 to 2.033) temperatures, and heat index (coefficient=1.094 to 1.456) (p values <0.001) (Table 7).

**Table 7:** Regression analysis between daily acute encephalopathy caseload and mean meteorological parameters for previous one, two, three, four, five, six, and seven days CI: confidence interval; hrs: hours; Kmph: kilometer per hour; mm: millimeter. * Correlation significant at the 0.05 level (2-tailed); ** Correlation significant at the 0.01 level (2-tailed).

Reference period for meteorological parameters	The regression coefficient for the climate/meteorological parameters (95% CI)						
	Maximum temperature (^0^C)	Minimum temperature (^0^C)	Humidity (%)	Wind speed (Kmph)	Precipitation (mm)	Sunshine duration (hrs)	Heat index
Previous one day (24 hours)	0.774** (0.355-1.193)	1.251** (0.674-1.828)	-0.068 (-0.177-0.039)	0.200 (-0.099-0.50)	-0.074 (-0.186-0.037)	1.112 (-0.106-2.331)	1.094** (0.674-1.514)
Previous two days (48 hours)	0.867** (0.427-1.306)	1.47** (0.855-2.086)	-0.073 (-0.186-0.039)	0.323* ( -0.011- 0.657)	-0.091 (-0.216-0.032)	1.355* (0.019-2.691)	1.218** (0.785-1.651)
Previous three days (72 hours)	0.889** (0.432-1.346)	1.626 ** (0.974-2.277)	-0.071 (-0.187-0.044)	0.409* (0.050-0.768)	-0.107 (-0.240-0.025)	1.520* (0.120-2.921)	1.287** (0.839-1.736)
Previous four days (96 hours)	0.894** (0.419-1.368)	1.757** (1.072-2.441)	-0.069 (-0.189-0.049)	0.514** (0.137-0.892)	-0.118 (-0.258-0.208)	1.610 * (0.155-3.064)	1.340** (0.875-1.805)
Previous five days (120 hours)	0.896** (0.404-1.388)	1.849 ** (1.131- 2.568)	-0.067 (-0.189-0.055)	0.635** (0.241-1.030)	-0.130 (-0.276-0.0155)	1.709* (0.199-3.220)	1.382** (0.898-1.866)
Previous six days (144 hours)	0.893** (0.382-1.403)	1.93 ** (1.1769-2.684)	-0.064 (-0.19-0.060)	0.742** (0.331-1.154)	-0.142 (-0.294-0.010)	1.829* (0.263-3.395)	1.418** (0.914-1.923)
Previous seven days (168 hours)	0.891** (0.361-1.422)	2.033 ** (1.242-2.823)	-0.062 (-0.191-0.066)	0.848** (0.423-1.273)	-0.153* (-0.312-0.005)	1.954* (0.322-3.587)	1.456** (0.928-1.984)

## Discussion

The 2019 outbreak investigation revealed that almost all children had early morning onset seizures and altered sensorium with hypoglycemia at admission. The multiorgan involvement (brain, liver, kidney, and muscles), hypoglycemia, ketonemia, and metabolic derangements involving fatty acids, tricarboxylic acid, ammonia, and ketone metabolism pathways indicated possible mitochondria dysfunction. The muscle histopathology and enzyme complex assay also supported mitochondrial pathology. The clinical and metabolic improvements with carnitine/coenzyme-Q therapy support this. Significant correlations between the number of admissions and the ambient temperatures (maximum and minimum) and the heat index were observed.

The past outbreaks of acute encephalopathy in Muzaffarpur (during 2011, 2013, and 2014) also occurred during the first two weeks of June. According to the reports, the median ages of children affected were 3.5-4 years and they had seizures (94-100%), unconsciousness (93-100%), decerebration (50%), tachycardia (80%), and tachypnea (80%). Hypoglycemia (62%), hyponatremia (90%), hypokalemia (5%), raised AST (30%), and raised blood urea (40%) with normal creatinine. No infectious cause was identified during these years. High case fatality (32-44%) was noted [2-4,5,15]. The majority of these children were from socially backward communities (59%) and scheduled castes (32%). Higher risk was observed among children from non-concrete houses (OR=1.93), parents with agriculture occupation (OR=2.12) and low education (OR=1.73), residence near litchi orchard (OR=3.31), and children who spent more time outdoors (OR=2.6) [4,15]. Abnormal urinary organic aciduria (89%) and plasma acylcarnitine profiles (90%) were observed during the 2014 outbreak. Higher associations with litchi consumption (OR=9.6), orchard visit (OR=6.0), and missing evening meals (OR=2.2) were noted. With documentation of hypoglycin A and/or MCPG in 44-64%, Lychee consumption was proposed as the factor for encephalopathy [5].

The reports for 2011, 2013, 2014, and 2019 indicated non-infective and metabolic aetiology for encephalopathy during the May-June months. Multiorgan (brain, liver, kidney, muscle and heart) involvement with abnormal CPK-MB, LDH, lactate, urinary organic aciduria and plasma acylcarnitine profiles were documented in these children. During the 2019 outbreak, ketonemia/ketonuria was documented in several cases, but with MCPG, hypoketonemia is expected [6]. The history of lychee intake was noted in 27% of cases in 2019. Many parents denied lychee intake by their children, as the local government campaigned against children consuming lychee after 2017. Urinary lychee metabolites detection may not correlate with fruit intake amount. In absence of the lethal dose (LD50) and effective dose (ED50) of lychee for humans, considering the doses for rats, about 300 lychees are to be consumed, which is difficult for young children [5,7,16,17]. Minimal involvement of children from upper socioeconomic strata does not support the lychee consumption hypothesis, as lychee is consumed by all.

The electron microscopy findings and reduced respiratory chain enzyme complexes improvements in metabolic parameters after carnitine/coenzyme-Q therapy are pointers toward potential secondary mitochondrial dysfunction. Seizures in children with carnitine/acylcarnitine deficiency have been reported [18].

During May-June, the Muzaffarpur climate becomes extremely hot (highest: 40-46^0^C, lowest 28-30^0^C) and humid (50-60%). More cases were observed when the highest temperature was above 40^0^C for some days [3]. A recent report by our group reported positive correlations between acute encephalopathy caseload (n=1318, during 2012-2020) with maximum (r=0.275) and minimum (r=0.306) temperatures and heat index (r=0.325) and negative correlation with humidity (r=−0.222) and rainfall (r=−0.183) during past 24 hours (all p < 0.05). The correlation was also consistent for these climate parameters for the past 48 and 72 hours before admission [19]. The significant positive correlation between acute encephalopathy caseload and ambient climate parameters (maximum and minimum temperatures and heat index) suggests a possible relationship, if not causative, a contributory factor for mitochondrial pathology.

Heat-related morbidity and mortality have been observed from the third day of high temperatures [8]. Mitochondrial dysfunction with reduced adenosine triphosphate (ATP) production, raised serum CPK, LDH, and transaminases have been documented with heat stress [9-11]. In animals, exposure above 39^0^C has shown nuclear chromatin condensation and mitochondrial alterations (reduced number of cristae membranes, membrane disruptions, and swelling) in the myocardium [12]. Cerebral oedema with changes in axons, nerves, glial cells, and vascular endothelium was observed in rats with exposure to ≥38^0^C [13,14]. Fatty-acid-oxidation defects in fibroblasts were noted with exposure to ≥41^0^C [20,21].

JE has been the leading infectious cause of acute encephalitis in children in India followed by scrub typhus and dengue, while the aetiology remains elusive in half of the cases. There is inter-state variation in the proportion of infectious aetiologies contributing to acute encephalitis in children. The aetiological profile is also changing with the introduction of the JE vaccination in selected areas. There is also clinical ambiguity in labelling the cases, in most scenarios the cases are clubbed as AES, which includes both encephalitis and encephalopathy, which makes it difficult to differentiate between these entities, whether a different neurological disorder or any combination. Despite these variations, the acute encephalopathy outbreak in children from Muzaffarpur and nearby areas remains a challenge, with limited success from infectious aetiological investigations.

Acute encephalopathy outbreaks have been reported from Vietnam and Bangladesh during the May-June months. The Bangladeshi children had fever (89%), seizures (82%), vomiting (64%), meningeal signs (85%), and altered sensorium (87%). Associations with lychee orchard exposure (age-adjusted OR (aOR)=2.3-4.1), staying near lychee orchard (aOR=1.3), and agriculture family (aOR=2) were noted [22-24]. In Vietnam, enteroviruses in CSF (35%), abnormal hypoglycin-A and MCPG (45%), and β-oxidation inhibition (40%) were noted, suggesting a mixture of infection and possible metabolic causes [25].

These indicate the ongoing debate and confusion regarding the aetiology across India and different neighbouring countries. The efforts for viral and other infectious aetiological isolation have been partially successful. Thus, there is a need to think beyond the infectious causes for finding the aetiology.

The study has several limitations. This study collected data during the response to the acute encephalopathy outbreak and not as a systematically designed study. The detailed clinical and nutritional assessment and investigations could not be undertaken for all patients and only a small number of patients could be subjected to detailed biochemical, metabolic, and mitochondria histopathology evaluation. It didn't include any control participant or community investigation to identify risk factors.

## Conclusions

The findings suggest the possible role of secondary mitochondrial dysfunction for acute encephalopathy in children from Muzaffarpur during the summer. Although the exact cause of mitochondrial dysfunction could not be established, heat stress with underlying malnutrition and micronutrient deficiencies may be possible explanations. Carefully designed studies using a robust methodology, markers of metabolic encephalopathy and mitochondrial derangements linked to the environmental heat stress indices, and individual-level factors (sociodemography, nutrition, and other contributors) are needed to confirm the association or causal linkages.
